# Development and implementation of an automated and highly accurate reporting process for NGS-based clonality testing

**DOI:** 10.18632/oncotarget.28429

**Published:** 2023-05-12

**Authors:** Sean T. Glenn, Phillip M. Galbo, Jesse D. Luce, Kiersten Marie Miles, Prashant K. Singh, Manuel J. Glynias, Carl Morrison

**Affiliations:** ^1^Department of Pathology, Roswell Park Comprehensive Cancer Center, Buffalo, NY 14263, USA; ^2^Department of Cancer Genetics and Genomics, Roswell Park Comprehensive Cancer Center, Buffalo, NY 14263, USA

**Keywords:** clonality, NGS, bioinformatics, molecular diagnostics, leukemia

## Abstract

B and T cells undergo random recombination of the VH/DH/JH portions of the immunoglobulin loci (B cell) and T-cell receptors before becoming functional cells. When one V-J rearrangement is over-represented in a population of B or T cells indicating an origin from a single cell, this indicates a clonal process. Clonality aids in the diagnosis and monitoring of lymphoproliferative disorders and evaluation of disease recurrence. This study aimed to develop objective criteria, which can be automated, to classify B and T cell clonality results as positive (clonal), No evidence of clonality, or invalid (failed). Using clinical samples with “gold standard” clonality data obtained using PCR/CE testing, we ran NGS-based amplicon clonality assays and developed our own model for clonality reporting. To assess the performance of our model, we analyzed the NGS results across other published models. Our model for clonality calling using NGS-based technology increases the assay’s sensitivity, more accurately detecting clonality. In addition, we have built a computational pipeline to use our model to objectively call clonality in an automated fashion. Collectively the results outlined below will have a direct clinical impact by expediting the review and sign-out process for concise clonality reporting.

## INTRODUCTION

**Figure 1:**
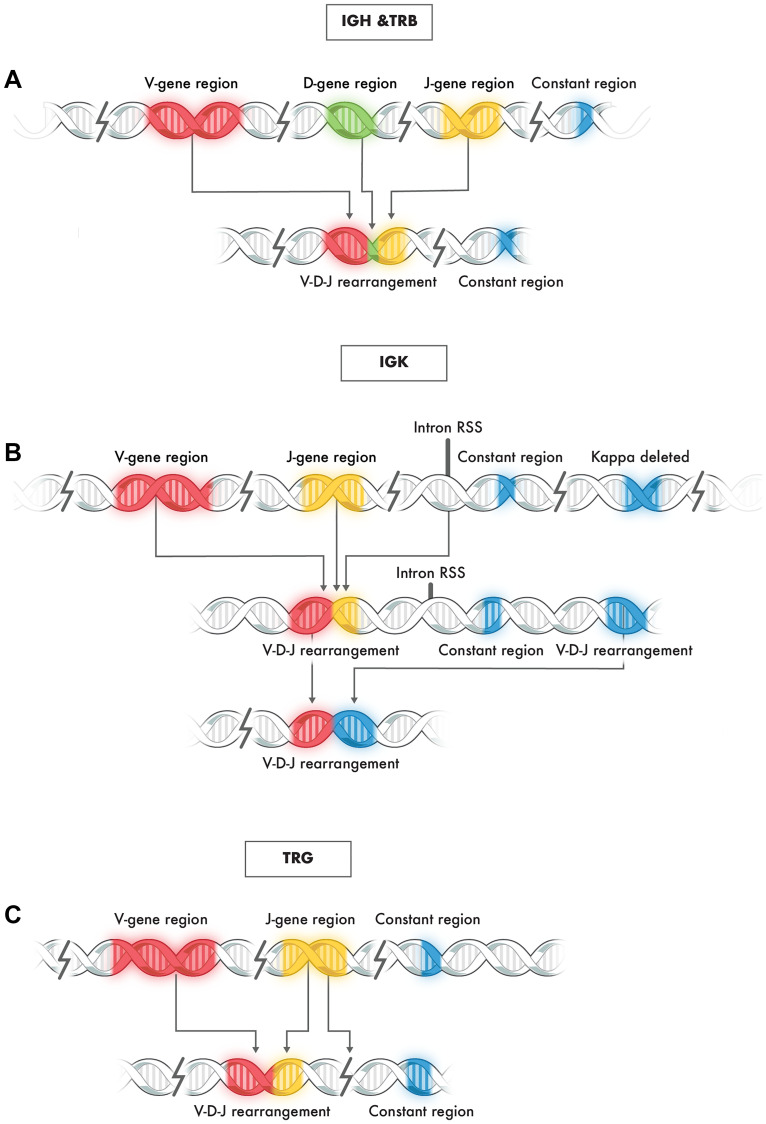
Typical rearrangement of genes at immunoglobulin loci IGH, TRB, IGK, and TRG. (**A**) Representative schematic of V-D-J rearrangement in IGH and TGB loci: (**B**) Representative schematic of V-J rearrangement of the IGK locus: (**C**) Representative schematic of V-J rearrangement of the TRG locus.

Assessment of clonality by evaluation of rearrangement of immunoglobulin loci and T cell receptors is an integral part of the diagnostic workup of lymphoproliferative diseases. Immunoglobulin loci are composed of multiple functionally related genes that are organized in clusters at specific chromosomal locations, including the heavy chain (IGH) gene locus on chromosome 14 (14q32.3) and kappa light chain (IGK) gene locus on chromosome 2 (2p11.2) for B cells, with T cell receptor rearrangement at the gamma (TRG) gene complex at 7p15-p14 and beta (TRB) gene complex at 7q34 for T cells [1–3]. The B and T cell rearrangements mentioned above do not include all such immune-related gene clusters but cover those most frequently used for clinical molecular purposes to interrogate genomic DNA-based rearrangement of these regions [4]. In cells other than lymphocytes, these gene segments are separated from one another by large segments of intervening DNA and remain in this germline state [1, 2, 4]. In contrast, both normal and neoplastic lymphocytes undergo random recombination of these gene complexes early in their development [5, 6]. During this process, various segments, or exons of specific genes, are shuffled and positioned in proximity to one another by the deletion of internal DNA sequences (Figure 1). The typical gene locus arrangement includes a variable, diversity, joining, and constant segment for which only some are functional.

Due to these rearrangements and subsequent recombination events, each B or T lymphocyte contains a uniquely rearranged B or T immunoglobulin receptor gene [7]. These rearrangements can be used in the molecular evaluation of clonality since the rearrangement profiles of neoplastic and benign lymphoproliferative conditions are different [7, 8]. Clonal proliferations are characterized by a homogeneous B or T cell receptor gene rearrangement profile. In contrast, polyclonal proliferations, such as reactive lymphocytic hyperplasia, contain B or T cells derived from multiple parental clones. Therefore, each B or T lymphocyte in this milieu contains a different V-J sequence, providing a heterogeneous B or T cell receptor gene rearrangement profile.

The current standard of practice for clonality evaluation includes flow cytometry and the evaluation of rearrangement of B- and T-cell receptors [9–11]. Flow cytometry and the traditional method of PCR and capillary electrophoresis for evaluation of rearrangement of B- and T-cell receptors are semi-quantitative and, by default, result in a subjective interpretation of clonal versus non-clonal [12–14]. With the advent of next-generation sequencing (NGS) technology, it is possible to achieve a quantitative result and apply an objective interpretation of clonal versus non-clonal [15].

**Figure 2:**
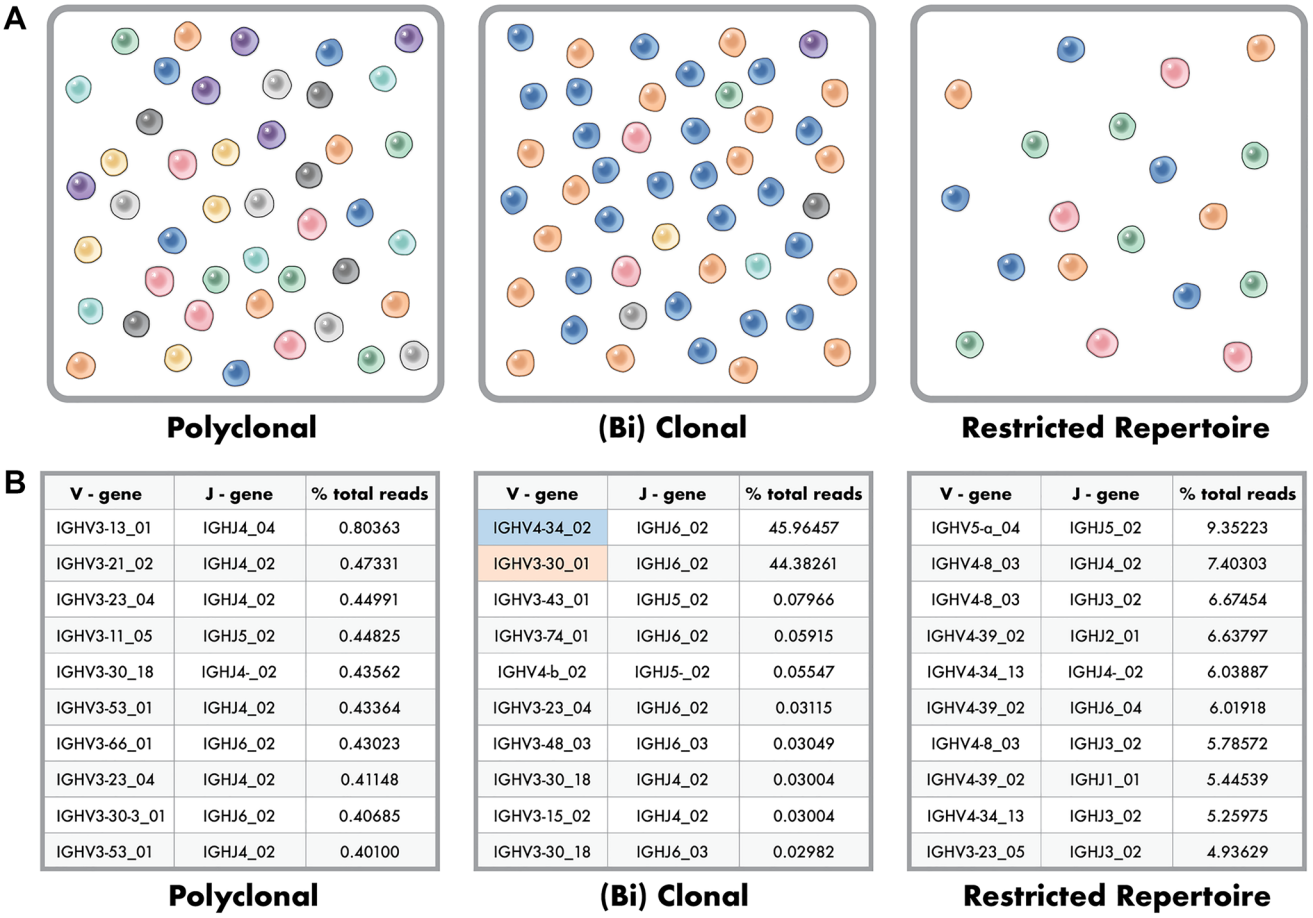
Polyclonal, Clonal and Restricted Repertoire Phenotypes in Clonality Testing. (**A**) Polyclonal phenotype (right image) where many distinctly different clones are represented evenly at very low frequency within the population. Clonal, or (bi) clonal phenotype (center image) where two dominant clones make up a large percent of the population with remaining clones below a percent frequency. Restricted repertoire (right image) where the number of unique clones is low artificially showing high percent for the available clones due to a paucity of B or T cells in the sample. (**B**) Example data for the top ten clones identified in polyclonal (right data), (bi) clonal (center data), and restricted repertoire (right data) phenotypes as depicted in 2a.

Enabling the application of NGS for clonality evaluation is the readily available commercial sources of primers and reagents for library production, such as the Invivoscribe LymphoTrack IGH FR1/2/3 Assay Panel [16, 17], LymphoTrack IGK Assay Panel [17, 18], LymphoTrack TRB Assay Panel [17, 19], and LymphoTrack TRG Assay Panel [17, 19], that can be sequenced on an Illumina MiSeq or Ion Torrent PGM. Likewise, available bioinformatic tools, such as the LymphoTrack Software, enable the processing of NGS-generated FASTQ files of B- and T-cell receptors that result in aligned clonotypes that can be ranked by relative dominance. In a sample with identifiable B- or T-lymphocytes by histological evaluation that is clinically consistent with a polyclonal process, the evaluation of clonality by NGS typically yields hundreds of individual unique V-J sequences, or so-called clonotypes, each with multiple reads due to PCR amplification [20, 21]. Due to potential alignment errors in the bioinformatics pipeline, merging individual sequences with 1% or fewer sequence differences is common, referred to as merged reads [22]. Nonetheless, polyclonal processes with identifiable B- or T-lymphocytes by histological evaluation yield many clonotypes, each that should represent less than 1% of all NGS reads for that sample (Figure 2A-Left Panel).

In contrast, a sample with a identifiable B- or T-cell lymphoproliferative process shows one to two (biallelic) dominant clonotypes, typically with a polyclonal background (Figure 2A-Center Panel). Between these two ends of the spectrum that are somewhat easy to classify are less distinctly classified scenarios, including samples with a paucity of B- or T-lymphocytes and analysis of B-cell clonality in a T-cell lymphoproliferative process and vice versa [15]. These latter scenarios represent a restricted B- or T-cell repertoire, or limited rearranged immunoglobulin loci for evaluation, with, for this specific sample, fewer overall clonotypes among all NGS reads (Figure 2A-Right Panel). The result is multiple clonotypes, some of which represent greater than 1% of all reads for that sample, but none of which is readily dominant to enable a distinct classification of clonality versus no evidence of clonality (Figure 2B).

There have been prior efforts to develop rule sets for this purpose to enable the distinction of clonality versus no evidence of clonality and to use the quantitative capabilities of NGS analysis [15]. Arcila et al. (2019), in their evaluation of IGH FR1/FR2/FR3 and IGK using LymphoTrack reagents for B-cell clonality, offered different criteria for clonality versus no evidence of clonality based upon the number of reads with samples having equal to or greater than 100,000 reads defining a dominant clone as equal to or greater than 2.5% of total reads and that this value is 10× the polyclonal background [23]. For samples with less than 100,000 but equal to or greater than 30,000 reads, a dominant clone was defined as equal or greater than 5% of total reads, and this value is 20× the polyclonal background [23]. A polyclonal background was not explicitly defined but was referred to as the 3rd most dominant clone [23]. For T-cell clonality, Nollet (2018) and Schumaker (2014) evaluated TRG by NGS; a dominant clone was defined as equal to or greater than 4% of total reads, and this value is 4.5× the polyclonal background [24, 25]. The polyclonal background was defined as the highest clone percent 2× less than the next most frequent clone in the ten most frequent merged clones. Neither Nollet (2018) nor Schumaker (2014) provided information about classification when the polyclonal background failed to have a minimum of one clone in the top 10 that was 2× less than the next most frequent clone [24, 25]. Likewise, the manufacturer and Arcila et al. do not define explicit criteria for a polyclonal background. Invivoscribe, the manufacturer of reagents in all these prior studies, recommends defining clonality as a dominant clone equal to or greater than 2.5% of total reads and that this value be 10× the third most frequent clone [23–25]. Furthermore, the manufacturer recommends applying this rule to IGH FR1/FR2/FR3, IGK, TRG, and TRB.

While we recognize that most cases evaluated for clonality can be accurately and efficiently defined by these initial rule sets, in our experience, there are many instances where the results are less than optimal including edge cases which require defining a stringent polyclonal background for accurate assessment of clonality (i.e., evidence of a restricted repertoire). In that regard, we set out to develop an algorithmic approach that does not allow for subjective interpretation and provides an optimal solution for most cases. To do so, we selected a relatively large cohort of cases previously classified by PCR/capillary electrophoresis represented by polyclonal, clonal, and bi-clonal (both B- and T-cell clonal) and evaluated them using the LymphoTrack NGS reagents on an Illumina MiSeq [17, 19]. These results were then evaluated for clonality versus no evidence of clonality using multiple approaches, including the manufacturer’s recommendation for IGH FR1/FR2/FR3, IGK, TRG, and TRB, that of Arcilia et al. 2019 for IGH FR1/FR2/FR3, and IGK, and that of Nollet et al. 2018 and Schumaker et al. 2014 for TRG and TRB, and our in-house custom design algorithm [23–25].

## RESULTS

### Criteria for assessing different models of determining clonality

The goal of this study was to develop objective criteria to classify B and T cell clonality results as positive (clonal), no evidence of clonality (NEC), or invalid (failed). Criteria were developed by testing multiple accuracy models using previously PCR/CE tested samples. Five of these six models were similar in using three primary endpoints of sample acceptance, clone frequency, and polyclonal background. In these five models, sample acceptance was based upon several reads.

For sample acceptance, the underlying premise for using the number of reads is to identify low-quality samples that could result in false-negative calls. The manufacturer’s recommendation (Invivoscribe, Inc) for the number of total sequencing reads is 20,000 at the sample level. Other peer-reviewed publications have set this value across a diverse range from 50,000 [23] for IGH FR1/FR2/FR3 and IGK to 1,000 total reads for TRG [24, 25].

Table 1 below summarizes the analysis for a clonal call by the manufacturer’s recommendations, prior studies [23–25], and our internal Roswell Park approach with more precisely defined criteria. More specifically, Table 1 outlines the criteria of various models for the read count threshold to fail a sample for analysis, an overview of the merge rule, and the criteria for defining a polyclonal background. Note that while the Roswell Park model has different criteria for IGH FR1/FR2/FR3 as compared to IGK, the results are combined to produce a final clonality assessment. In any of these models for B cell determination, any single assay of IGH FR1, IGH FR2, IGH FR3, or IGK can meet the criteria for a clonal call, and the overall result for the case is positive or clonal due to the related biological events of heavy and light chain rearrangement. While TRG and TRB are not distinct biological-related events, a clonal result for either assay is considered a positive or clonal result at the case or patient level. As stated in the introduction of this validation, we had gold standard samples for TRG but not for TRB, as the latter was not part of our prior clinical testing.

**Table 1:** Criteria of various models for the read count threshold to fail a sample for analysis, an overview of the merge rule, and the criteria for defining a polyclonal background

Model	Clone % criteria	Polyclonal background criteria	Clonal result	No evidence of clonality result	Other results	Fail result
Invivoscribe	2.50%	10×	clonal if passes criteria for both clone % and polyclonal background	Not clonal	None	<20,000 reads
MSK (Arcila et al. 2019 B cell)	5% for <100,000 reads	20×		Not clonal or oligoclonal	oligoclonal if ≥3 dominant clones and >100,000 reads	<30,000 reads
	2.5% for ≥100,000 reads	10×				
Nollet et al. 2018 and Schumaker et al. 2014 for TRG	4%	4.5×		No clone with % reads greater than 2%	Polyclonal with minor clonal rearrangements if does not meet clonal or polyclonal criteria	<1,000 reads
Roswell Park FR1, FR2, FR3	any top two clones combined ≥30.0% without regard to polyclonal background or >2.49% with polyclonal background rule	10×	Top two clones combined ≥30.0% without regard to polyclonal background, or less than 50% but greater than 2.5% and 10× polyclonal background	Does not meet clonal criteria	None	<20,000 reads
Roswell Park IGK, TRG, TRB	any top two clones combined ≥50.0% without regard to polyclonal background or >4.99% with polyclonal background rule	10×	Top two clones combined ≥30.0% without regard to polyclonal background, or less than 30% but greater than 4.99% and 10× polyclonal background	Does not meet clonal criteria		

### An improved model for B cell clonality determination

To assess our ability to call B cell clonality using our previously defined thresholds, we included 36 samples (25 clonal, 11 polyclonal) which were a mixture of high-and-low quality DNA (21 BM or BLD, 15 FFPE). As previously described (Table 1), the four different models were tested for accuracy that was applied in the same fashion for both B and T cell clonality. In addition, the Nollet/Schumaker model was also tested with a more typical cut-point of total reads at 20,000 rather than 1,000 and is referred to as the modified N/S model [24, 25].

When assessing the concordance to gold standard CE/PCR results across all models, four samples were consistently called false negatives (Supplementary Table 1). Three of these four cases were either blood or bone marrow post-transplant with no evidence of recurrent disease by pathological evaluation, flow cytometry, or cytogenetics. The fourth case was a Hodgkin lymphoma, a tumor type that typically would not be expected to have immunoglobulin receptor rearrangement. Given that all models for these cases were a call of polyclonal or NEC for all assays, there is a consideration that the gold standard clonal calls by CE/PCR were incorrect and were therefore excluded from the final evaluation (Table 2). One additional case was called a false negative by the Invivoscribe and MSK models and correctly called by the Nollet/Schumaker and Roswell Park models. The Nollet/Schumaker model called the only false positive call across all models. When the four cases that were false-negative across all models are removed from consideration and the gold standard results are updated, the RP model has 100% sensitivity and specificity as well as PPV and NPV (Table 2).

**Table 2:** Summary of results for different models of determining b cell clonality

Model	#TP	#TN	#FP	#FN	Sensitivity	Specificity	PPV	NPV
Invivoscribe	20	11	0	1	95.20%	100.00%	100.00%	91.70%
Arcila et al. (2019) for B cell/MSK	20	10	0	1	95.20%	100.00%	100.00%	90.90%
Nollet et al. (2018) and Schumaker et al. (2014) for TRG	21	10	1	0	100.00%	90.90%	95.50%	100.00%
Roswell Park FR1, FR2, FR3, IGK	21	11	0	0	100.00%	100.00%	100.00%	100.00%

### An improved model for T cell clonality determination

To assess our ability to call T cell clonality for TRG, we included 31 samples (21 clonal, 10 polyclonal) with a mixture of high-and-low quality (14 BM or BLD, 17 FFPE). As previously described (Table 1), the four different models were tested for accuracy of T cell clonality in the same fashion as for B cell clonality.

Like the B cell clonality data, there were three false negative samples across all models that would suggest the gold standard result of clonal could be wrong (Supplementary Table 2). One case, 01659, was a surgical pathology biopsy of an Epstein Barr Virus mucocutaneous ulcer with a B cell phenotype, abundant histiocytes, and a paucity of T cells. The second case, 03599, was a bone marrow of an allogenic post-transplant CLL that, upon H&E evaluation, was described as a suboptimal specimen that was paracellular but consistent with post-transplant recovery. Matching flow cytometry and peripheral blood exam showed no evidence of CLL. The third case, 02319, was a surgical pathology biopsy of a Hodgkin lymphoma, which typically does not have B or T cell rearrangement. The sum of these clinical comparisons and current results across all models would support that the gold standard clonal calls were incorrect and have therefore been excluded in the results (Table 3).

**Table 3:** Summary of results for different models of determining T cell clonality

Model	#TP	#TN	#FP	#FN	Sensitivity	Specificity	PPV	NPV
Invivoscribe	10	10	0	8	55.60%	100.00%	100.00%	55.60%
Arcila et al. (2019) for B cell/MSK	10	10	0	8	55.60%	100.00%	100.00%	55.60%
Nollet et al. (2018) and Schumaker et al. (2014) for TRG	14	8	2	4	77.80%	80.00%	87.50%	66.70%
Roswell Park TRG & TRB	17	8	2	1	94.40%	80.00%	89.50%	88.90%

In contrast to the results for B cell accuracy, those for T cell clonality showed more variance across the four different models. The Invivoscribe and MSK models were identical across all samples for TRG, but with a high number of false negative calls resulting in a suboptimal sensitivity and NPV of 55.6% for both values, but with specificity and PPV of 100.0% (Table 3 and Supplementary Table 2). The NS and RP models had higher sensitivity of 77.8% and 94.4%, respectively, and very comparable values for PPV of 87.5% and 89.5%, respectively. The Roswell Park model had the highest NPV of 88.9%, compared to 66.7% for NS and 55.6% for the Invivoscribe and MSK models. While we did not have gold standard data for the TRB assay, the results were in line with TRG as the Invivoscribe and MSK models showed similar results and fewer clonal calls than the NS and RP models (Supplementary Table 3).

### Automation of clonality calling

**Figure 3:**
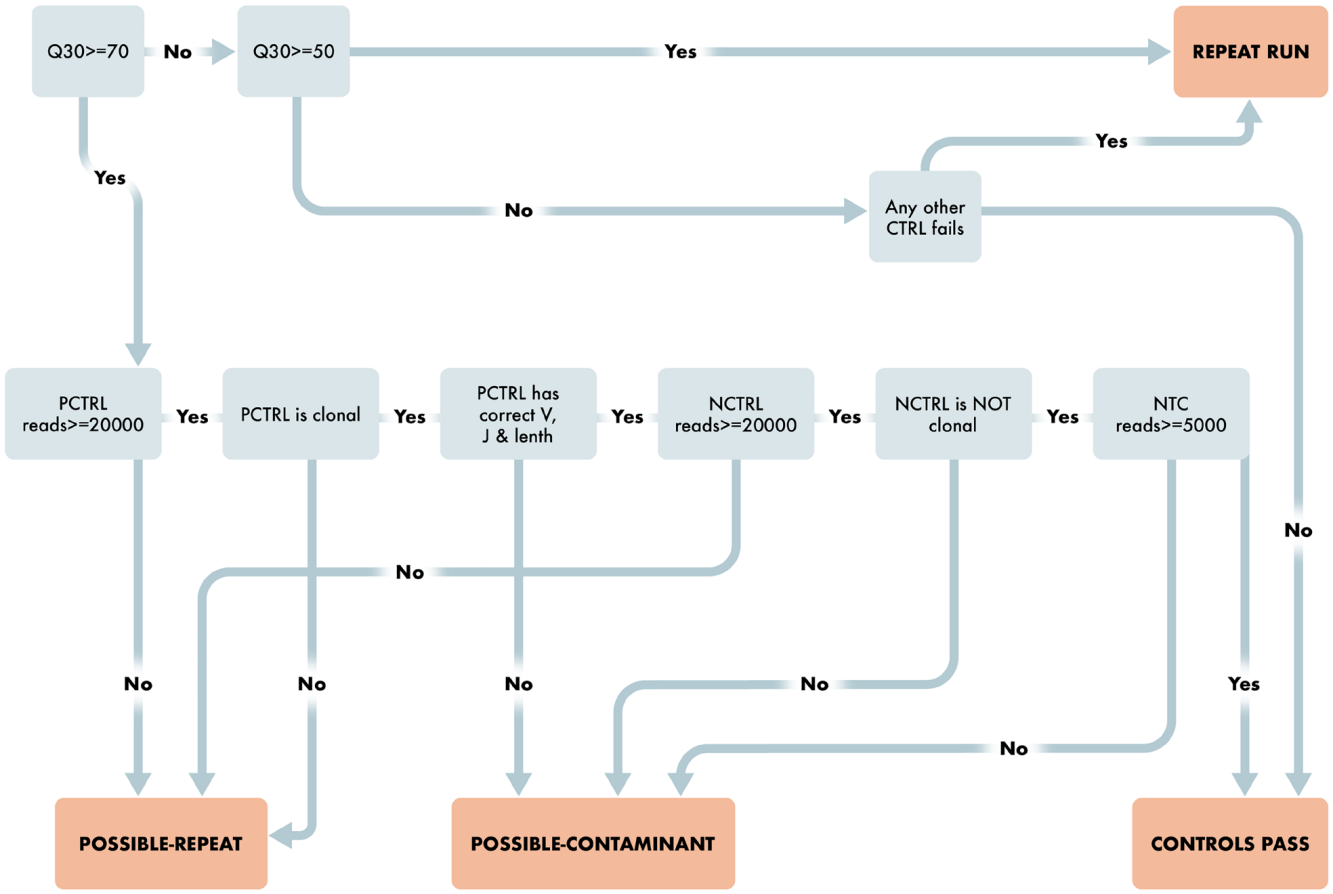
Workflow for Run and Control Level QC Characteristics. Schematic representing the steps required for the clonality algorithm to pass a run. Starting with run level QUAL evaluation and then reviewing the appropriate Positive and Negative control requirements to pass the run.

Beyond our goal of creating criteria that lend to more accurate reporting, we also wanted to build an automated computational pipeline that empirically reviews run, control, and sample level quality and calls clonality without subjective interpretation. The algorithm and software for automatically capturing fastq files and generating them into up-loadable data to a clinical EHR system are of keen interest to our molecular lab and ordering physicians. The functional software that contains this algorithm also initially interacts with the sequencing QC file to make sure an adequate Q30 score is met and runs the Invivoscribe supplied positive and negative control through the algorithm to “Pass” the run prior to analyzing the clinical samples. This step is paramount to ensure that the run level performance is adequate for accurate reporting of clinical results and is outlined in Figure 3.

**Figure 4:**
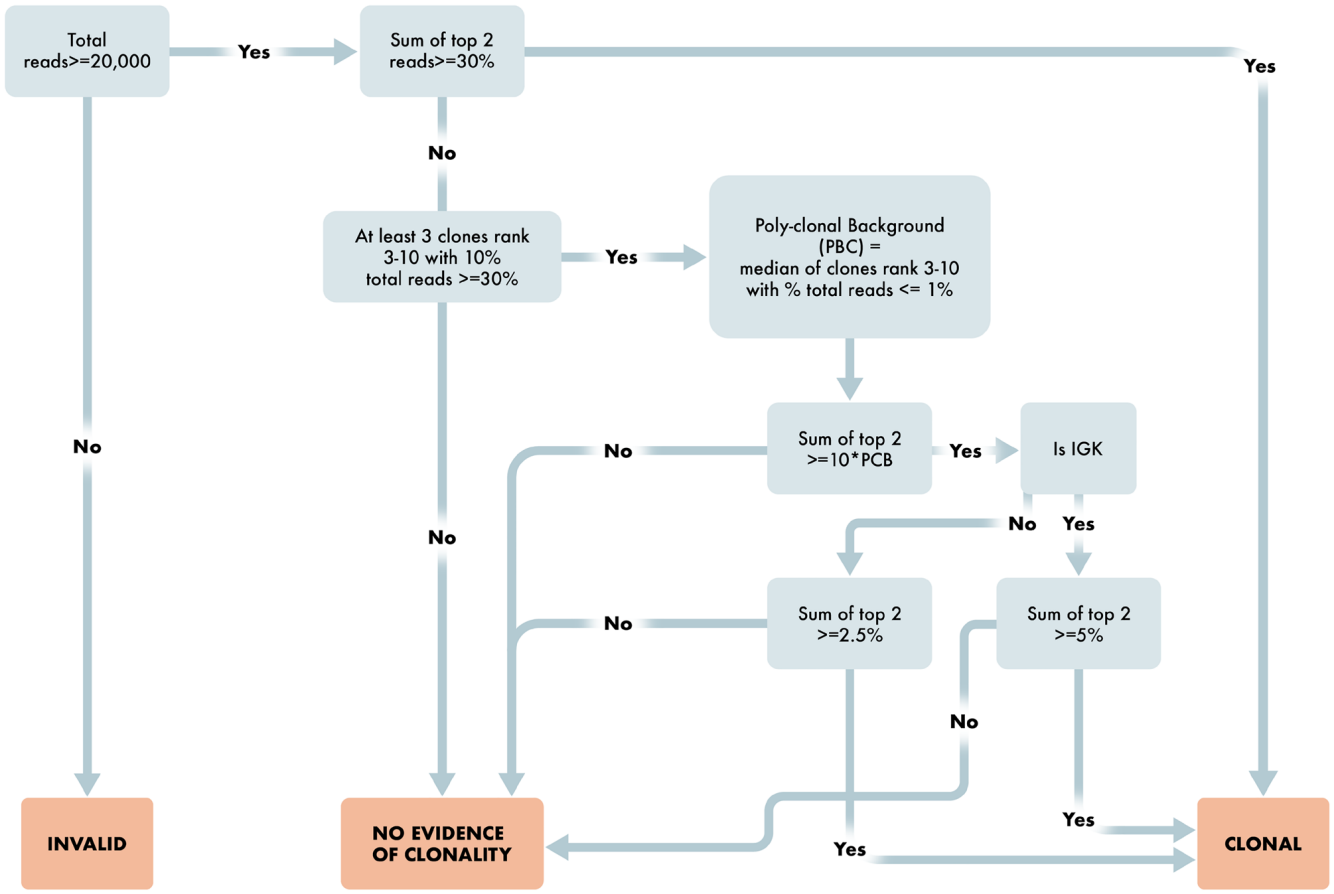
Workflow for determination of B cell clonality. Overview schematic of steps required for determination of clonality status in B cell clonality algorithm. Included in these steps are read requirements, percent dominant clone and polyclonal determining factors for appropriate reporting of B cell clonality status.

**Figure 5:**
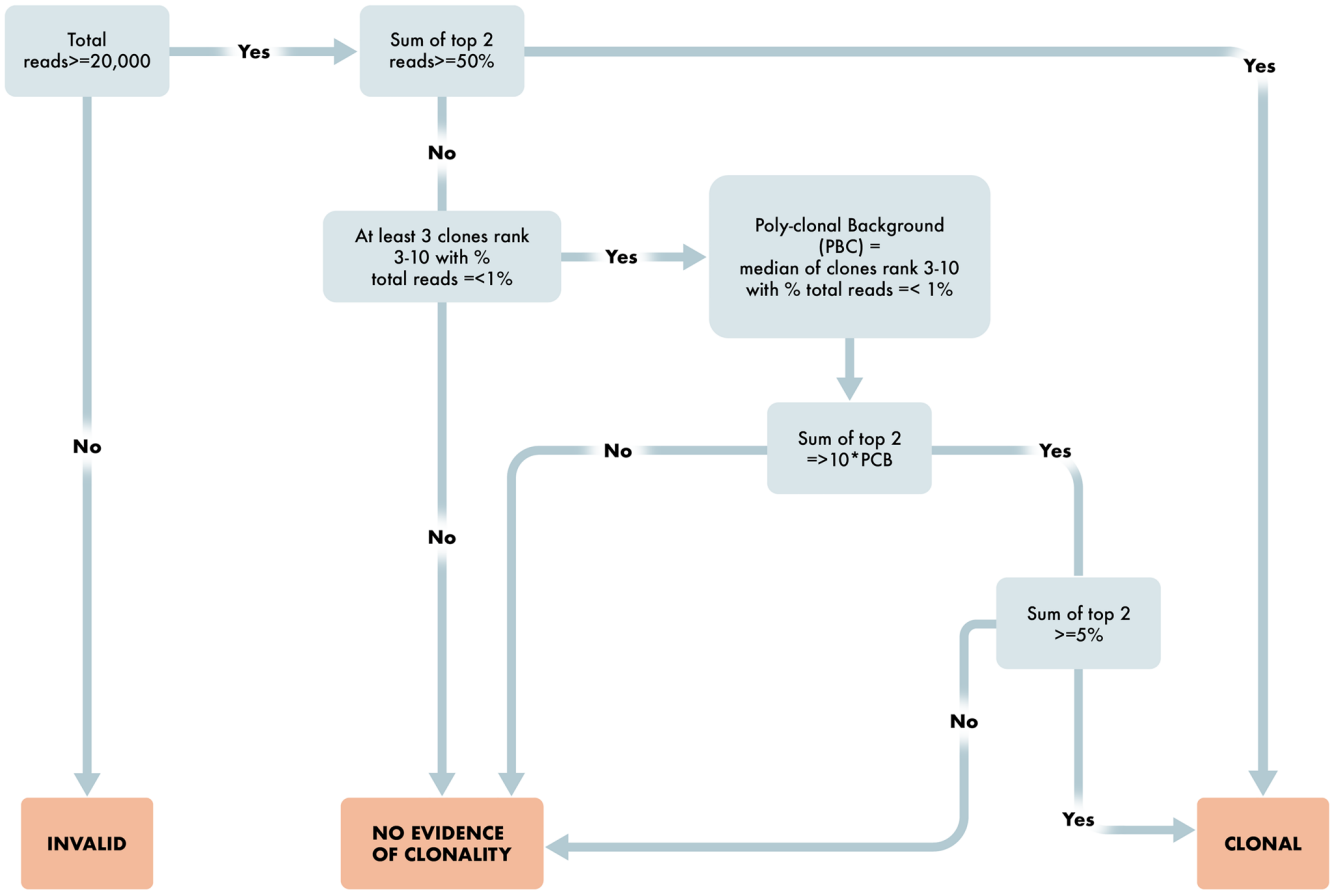
Workflow for determination of T cell clonality. Overview schematic of steps required for determination of clonality status in T cell clonality algorithm. Included in these steps are read requirements, percent dominant clone and polyclonal determining factors for appropriate reporting of T cell clonality status.

After run and control level QC, the algorithm determines results for each case depending on the number of reads per sample, percent of total reads for each clone, and comparison to background clones. The workflow is provided in detail as steps 1 through 9 below and in Figures 4 and 5.

The algorithm for B cell Clonality is, for each assay of IGH_FR1, IGH_FR2, IGH_FR3, and IGK, is as follows:

1. Are the number of sequencing reads ≥20,000 total reads? If no, then Invalid result. If yes, proceed to 2.
2. Are the top two merged reads for any assay ≥ 30%. If yes, then B cell Clonal. If no, proceed to 3.
3. Are there at least three clones ranked three through ten with percent total reads ≤1% (≤1.25% for IGK)? If no, then No Evidence of Clonality. If yes, the Poly-clonal Background is defined as the median of these reads; proceed to 4.
4. Are the top two merged reads ≥10× the Poly-clonal Background? If no, then No Evidence of Clonality. If yes, proceed to 5.
5. Is the assay IGK? If yes, proceed to 6. If no, proceed to 7.
6. Is the sum of the top two reads ≥5%? If yes, then B cell Clonal. If no, then No Evidence of Clonality.
7. Is the sum of the top two reads ≥2.5%? If yes, then B cell Clonal. If no, then No Evidence of Clonality.
8. If any assay is B cell Clonal, then the result for the test is B cell Clonal.

The algorithm for T cell Clonality is, for each assay of TRB, TRG, as follows:

1. Are the number of sequencing reads ≥20,000 total reads? If no, then Invalid result. If yes, proceed to 2.
2. Are the top two merged reads for any assay ≥ 50%. If yes, then T cell Clonal. If no, proceed to 3.
3. Are there at least three clones ranked three through ten with percent total reads ≤1.25%? If no, then No Evidence of Clonality. If yes, the Poly-clonal Background is defined as the median of these reads; proceed to 4.
4. Are the top two merged reads ≥10× the Poly-clonal Background? If no, then No Evidence of Clonality. If yes, proceed to 5.
5. Is the sum of the top two reads ≥5%? If yes, then T cell Clonal. If no, then No Evidence of Clonality.

If any assay is T cell Clonal, then the result for the test is T cell Clonal.

## DISCUSSION

Our approach to developing an algorithmic assessment of clonality using NGS differed from prior approaches. First, we took into consideration that the analysis of IGH FR1/FR2/FR3 has some distinct inherent differences from IGK, TRG, and TRB. These differences are primarily related to the depth of the polyclonal background for IGH FR1/FR2/FR3 as compared to IGK, TRG, and TRB. Due to the extensively more possible recombination events for IGH FR1/FR2/FR3 than for IGK, TRG, and TRB, the polyclonal background is much more likely to be more evenly distributed across a more significant number of unique reads. This results in the percent total reads of individual clones in the top 10 merged reads typically having a polyclonal background with a lower median value for IGH FR1/FR2/FR3 compared to IGK, TRG, and TRB. As a result of this higher V-J diversity for IGH FR1/FR2/FR3, the threshold for defining a dominant clone(s) as percent total reads can be lower compared to IGK, TRG, and TRB.

Another significant difference in our approach compared to others was to define a polyclonal background more precisely with supporting data and to apply rules when these criteria are not met. In every run, Invivoscribe supplies a negative control for which no clone should have a value greater than 1% of total reads, or else that run is considered a failed run. In our study of B and T cell clonality, 64 negative control replicates across all assays were performed, with all observed values for percent total reads being less than 1%. Nollet et al. (2018), in their analysis of TRG for ten healthy donors, showed that the most frequent read across all samples had an average frequency of 1.03% ± 0.79% (mean ± standard deviation) with a range from 0.15% to 2.10% with all samples showing a polyclonal pattern. In that regard, we defined a polyclonal background as all clones in the ten most frequent merged reads having a value of less than or equal to 1% of total reads for IGH FR1, FR2, and FR3, and 1.25% for IGK, TRB, and TRG, and that at least 3 such clones must be present. By applying the rule set, there were instances where a polyclonal background was not present by these criteria. In some cases, with a very high percent of clonal B or T cells, we observed three or fewer merged reads, and we arbitrarily defined these cases as clonal if the top two clones combined have a percent total reads equal to or greater than 50%. At the other extreme, a sample with few B or T cell rearranged alleles will have a restricted repertoire with ten or more merged reads, most of which have a percent total reads greater than 1.0%. These cases do not meet the requirements for a polyclonal background, and in our validation, we did not observe any examples where the top two clones combined have a percent total reads equal to or greater than 50% and were classified as evidence of no clonality. As the NGS clonality field evolves, it would likely be optimal if these cases are defined as a restricted repertoire, as this would be more meaningful clinically. Additional studies of prior cases referred to as oligoclonal by capillary electrophoresis should provide more data around this issue.

Another point of analysis that can be construed to be clinically meaningful is monoclonal versus bi-clonal. One variable in this approach for which standardization of the process would have value is a more defined criteria for how similar reads are merged into a single read. The manufacturer recommends that the top 10 most frequent reads be merged with other top 500 reads if they have two or fewer base pair mismatches. Arcila et al. (2019) did not precisely define their process of merging, but the best interpretation is that all clones that differ by less than five nucleotides are merged [23]. The most significant and likely most frequent impact of merging is two dominant clones with similar sequence identity being referred to as monoclonal rather than biclonal and future testing for recurrence disease based upon sequence identity not taking into consideration the impact of this process.

Utilizing these discrete differences for determining clonality in our gold standard sample sets allowed us to have optimal sensitivity, specificity, PPV and NPV for B cell clonality calling, which was superior to the other pipelines evaluated in parallel. Furthermore, in our T cell clonality testing, we substantially increased our sensitivity and NPV, which allowed for the calling of far more true positives (fewer false negatives) compared to other modalities. Beyond strengthening the ability to determine clonality accurately, we also implemented our calling algorithm into a software solution whereby run, control, and sample level QC and subsequent clonality calling are fully automated with zero need for subjective interpretation. Integrating this software solution on the front end to the MiSeq sequencer output files and on the back end to our custom laboratory information management system (LIMS) allows for seamless data transfer and results. Furthermore, our custom LIMS has direct reporting to the hospital EHR system post sign-out of final report in the LIMS system, fully automating the order through reporting process in the laboratory.

In conclusion, we have developed a fully automated calling algorithm for determining B and T cell clonality from NGS data, with greater sensitivity than previously developed models. As implemented in our software suite, this algorithm allows for direct movement of sequencing data, through the QC and analysis process with reporting clonality results without any subjective interpretation or evaluation. This process allows for standardization and efficiency leading to faster reporting of data in NGS-based clonality testing, which is paramount for clinical testing laboratories to maintain their high quality in reporting.

## MATERIALS AND METHODS

### Gold standard specimens

Remnant DNA from prior clinical testing using a PCR/CE-based clonality assay was available for use as gold standards for this study. All DNA material was approved for assay development by the internal review board at Roswell Park Comprehensive Cancer Center (BDR-128520). Samples chosen for comparison of prior results included monoclonal and polyclonal results based upon IGH FR1, IGH FR2, IGH FR3, IGK-A, and IGK-B, and were representative of the most common previously tested sample types of bone marrow (BM) aspirates, blood (BLD), and formalin-fixed paraffin-embedded (FFPE) tissue. This initial analysis for B cell clonality was based upon the IGH FR1/2/3 and IGK (kappa) BioMed-2 primer design with IGH-FR1 products of 310–360 bp, IGH-FR2 250–295 bp, and IGH-FR3 60–150 bp, IGK-A 120–160 bp, 190–210 bp, and 260–300 bp, and IGK-B 210–250 bp, 270–300 bp, and 350–390 bp. A prior positive result, or monoclonal, was defined as a discrete PCR peak detected in at least one IGH or IGK primer whose height was 300% (minimum 3.0 ratio) of the highest peak height in a polyclonal background. A PCR/CE negative result, or polyclonal, was defined as an absence of a discrete dominant peak with PCR products of varying lengths constituting a normal Gaussian distribution.

Prior testing for T-cell clonality was based upon an evaluation of the T-cell receptor-γ (gamma) gene complex (TRG) and did not include the T-cell receptor-B (beta) gene complex (TRB). The test was designed as two primer pools (MI and MII) with products ranging in size from 170–230 bp. Similar to B cell clonality, a discrete PCR peak detected in either MI or MII whose height was 300% (minimum 3.0 ratio) of the height of the highest peak in a polyclonal background was required for a TRG clonal call. Likewise, a PCR/CE negative result, or polyclonal, for TRG was defined as an absence of a discrete dominant peak with PCR products of varying lengths constituting a normal Gaussian distribution.

### Assessment of clonality by next-generation sequencing

Using 100 ng DNA input for each target, NGS-based B&T cell clonality testing was performed using the commercially available Invivoscribe LymphoTrack assay (Invivoscribe, Inc.) [3, 17, 19], including vendor-supplied positive and negative control samples. This assay targets the IGH (FR1, FR2, and FR3) and IgK genes in B cells and the TRG and TRB genes within T cells. Individual PCR reactions were performed for each target using multiple master-mixes containing the appropriate primers with barcoded sequencing adaptors for multiplexing capabilities. Post amplification, libraries were purified using AMPure XP bead-based methods (Agencourt, Inc.) and quantitated using qPCR (KAPPA, inc.). Quantitated libraries were pooled in an equimolar concentration of 4 nM and subsequently diluted to 12–20 pM for loading onto a 2 × 300 cycle MiSeq flow cell (Illumina, Inc.), according to the manufacturer’s specification.

### Data analysis

Sequencing data were analyzed using LymphoTrack MiSeq Software version 2.4.3, running in a Docker container, which allowed for the development and integration of the analysis into an automated process, further described in the results section. In addition, specific quality thresholds further discussed in the Results section were defined at the run, control, and sample level and implemented into a specific software process for automated, concise rule reporting of clonality, alleviating the variability sometimes associated with subjective interpretation of results.

## SUPPLEMENTARY MATERIALS



## Abbreviations

NGS: Next Generation Sequencing
PCR: Polymerase Chain Reaction
CE: Capillary Electropheresis
IGH: Immunoglobulin Heavy Chain
IGK: Immunoglobulin Kappa
TRG: T-cell Receptor Gamma
TRB: T-cell Receptor Beta
FR(1-3): Frame Work (1-3)
NEC: No Evidence of Clonality
