# Supplementary material for: Development and implementation of an automated and highly accurate reporting process for NGS-based clonality testing
Source: Oncotarget. 2023 May 12;14:450–61. doi: 10.18632/oncotarget.28429 (PMC10178459; doi:10.18632/oncotarget.28429)
Supplement: Supplementary file 1 [file oncotarget-14-28429-s001.pdf]

Development and implementation of an automated and highly accurate reporting process for NGS-based clonality testing

SUPPLEMENTARY MATERIALS

Supplementary Table 1: Results of B cell accuracy for 4 models of clonality results

Case	Specimen Type	Flow Cell	Barcode	Prior B Cell Result	Invivoscribe Call	Invivoscribe Result	MSK Call	MSK Result	NS Call	NS Result	RP Call	RP Result
03014	BLD	JP9L4	ID15	Clonal	NEC	FN[*]	Polyclonal	FN[*]	Polyclonal	FN[*]	NEC	FN[*]
00720	BM	JP9L4	ID05	Clonal	NEC	FN[*]	Polyclonal	FN[*]	Polyclonal	FN[*]	NEC	FN[*]
03599	BM	JP9L4	ID09	Clonal	NEC	FN[*]	Polyclonal	FN[*]	Polyclonal	FN[*]	NEC	FN[*]
02319	FFPE	JMK3H	ID10	Clonal	NEC	FN[*]	Polyclonal	FN[*]	Polyclonal	FN[*]	NEC	FN[*]
01659	FFPE	JP9L4	ID13	Clonal	NEC	FN	Polyclonal	FN	Clonal	TP	Clonal	TP
01493	BM	JMKFL	ID14	Polyclonal	NEC	TN	Failed	Failed	Clonal	FP	NEC	TN
02090	BM	JMKGY	ID23	Polyclonal	NEC	TN	Polyclonal	TN	Polyclonal	TN	NEC	TN
02189	BM	JMKGY	ID16	Polyclonal	NEC	TN	Polyclonal	TN	Polyclonal	TN	NEC	TN
03017	BM	JLLTN	ID03	Polyclonal	NEC	TN	Polyclonal	TN	Polyclonal	TN	NEC	TN
02029	BM	JMKFW	ID07	Polyclonal	NEC	TN	Polyclonal	TN	Polyclonal	TN	NEC	TN
02942	FFPE	JML7N	ID08	Polyclonal	NEC	TN	Polyclonal	TN	Polyclonal	TN	NEC	TN
02961	FFPE	JMKFL	ID07	Polyclonal	NEC	TN	Polyclonal	TN	Polyclonal	TN	NEC	TN
03060	FFPE	JMKFW	ID01	Polyclonal	NEC	TN	Polyclonal	TN	Polyclonal	TN	NEC	TN
03040	FFPE	JML7N	ID09	Polyclonal	NEC	TN	Polyclonal	TN	Polyclonal	TN	NEC	TN
00338	FFPE	JMKGY	ID20	Polyclonal	NEC	TN	Polyclonal	TN	Polyclonal	TN	NEC	TN
01002	FFPE	JMKGY	ID01	Polyclonal	NEC	TN	Polyclonal	TN	Polyclonal	TN	NEC	TN
03972	BLD	JMJYB	ID02	Clonal	Clonal	TP	Clonal	TP	Clonal	TP	Clonal	TP
01464	BLD	JP9L4	ID04	Clonal	Clonal	TP	Clonal	TP	Clonal	TP	Clonal	TP
03101	BLD	JMKFW	ID04	Clonal	Clonal	TP	Clonal	TP	Clonal	TP	Clonal	TP
01859	BLD	JMK3H	ID07	Clonal	Clonal	TP	Clonal	TP	Clonal	TP	Clonal	TP
01542	BLD	JMKFL	ID11	Clonal	Clonal	TP	Clonal	TP	Clonal	TP	Clonal	TP
01520	BM	JMK3H	ID04	Clonal	Clonal	TP	Clonal	TP	Clonal	TP	Clonal	TP
01858	BM	JMJYB	ID25	Clonal	Clonal	TP	Clonal	TP	Clonal	TP	Clonal	TP
00726	BM	JLLTN	ID25	Clonal	Clonal	TP	Clonal	TP	Clonal	TP	Clonal	TP
01758	BM	JMJYB	ID23	Clonal	Clonal	TP	Clonal	TP	Clonal	TP	Clonal	TP
03584	BM	JMJYB	ID27	Clonal	Clonal	TP	Clonal	TP	Clonal	TP	Clonal	TP
02614	BM	JP9L4	ID08	Clonal	Clonal	TP	Clonal	TP	Clonal	TP	Clonal	TP
02306	BM	JLLTN	ID02	Clonal	Clonal	TP	Clonal	TP	Clonal	TP	Clonal	TP
02088	BM	JML7N	ID15	Clonal	Clonal	TP	Clonal	TP	Clonal	TP	Clonal	TP
01737	FFPE	JMKFL	ID12	Clonal	Clonal	TP	Clonal	TP	Clonal	TP	Clonal	TP
01228	FFPE	JMK3H	ID01	Clonal	Clonal	TP	Clonal	TP	Clonal	TP	Clonal	TP
02884	FFPE	JML7N	ID13	Clonal	Clonal	TP	Clonal	TP	Clonal	TP	Clonal	TP
01531	FFPE	JP9L4	ID12	Clonal	Clonal	TP	Clonal	TP	Clonal	TP	Clonal	TP
00935	FFPE	JP9L4	ID06	Clonal	Clonal	TP	Clonal	TP	Clonal	TP	Clonal	TP
01539	FFPE	JP9L4	ID07	Clonal	Clonal	TP	Clonal	TP	Clonal	TP	Clonal	TP
03281	FFPE	JML7N	ID10	Clonal	Clonal	TP	Clonal	TP	Clonal	TP	Clonal	TP

[*]Excluded from analysis

Summary	Invivoscribe Result	MSK Result	NS Result	RP Result
Sum FN	1	1	0	0
Sum TN	11	10	10	11
Sum TP	20	20	21	21
Sum FP	0	0	1	0
Sensitivity	95.2%	95.2%	100.0%	100.0%
Specificity	100.0%	100.0%	90.9%	100.0%
PPV	100.0%	100.0%	95.5%	100.0%
NPV	91.7%	90.9%	100.0%	100.0%

Supplementary Table 2: Results of T cell accuracy for 4 models of clonality results for TRG

Case	Specimen Type	Flow Cell	Barcode	Prior T Cell Result	Invivoscribe Call	Invivoscribe Result	MSK Call	MSK Result	NS call	NS Result	RP Call	RP Result
02029	BM	JP9L4	ID14	Clonal	Polyclonal	FN	Polyclonal	FN	Polyclonal	FN	Clonal	TP
01520	BM	JMK3H	ID05	Clonal	Polyclonal	FN	Polyclonal	FN	Polyclonal	FN	Clonal	TP
01464	BLD	JP9L4	ID04	Clonal	Polyclonal	FN	Polyclonal	FN	Polyclonal	FN	Clonal	TP
01659	FFPE	JMKL4	ID23	Clonal	Polyclonal	FN*	Polyclonal	FN*	Polyclonal	FN*	NEC	FN*
01531	FFPE	JMKL4	ID22	Clonal	Polyclonal	FN	Polyclonal	FN	Clonal	TP	NEC	FN
03599	BM	JMKL4	ID19	Clonal	Polyclonal	FN*	Polyclonal	FN*	Polyclonal	FN*	NEC	FN*
02319	FFPE	JMK3H	ID11	Clonal	Polyclonal	FN*	Polyclonal	FN*	Polyclonal	FN*	NEC	FN*
00935	FFPE	JMKL4	ID15	Clonal	Polyclonal	FN	Polyclonal	FN	Clonal	TP	Clonal	TP
01493	BM	JML7N	ID14	Clonal	Polyclonal	FN	Polyclonal	FN	Clonal	TP	Clonal	TP
03014	BLD	JMKL4	ID27	Clonal	Polyclonal	FN	Polyclonal	FN	Clonal	TP	Clonal	TP
01228	FFPE	JMK3H	ID02	Clonal	Polyclonal	FN	Polyclonal	FN	Polyclonal	FN	Clonal	TP
02090	BM	JMKGY	ID25	Polyclonal	Polyclonal	TN	Polyclonal	TN	Polyclonal	TN	NEC	TN
01542	BLD	JML7N	ID11	Polyclonal	Polyclonal	TN	Polyclonal	TN	Clonal	FP	Clonal	FP
01737	FFPE	JML7N	ID12	Polyclonal	Polyclonal	TN	Polyclonal	TN	Polyclonal	TN	NEC	TN
02884	FFPE	JML7N	ID13	Polyclonal	Polyclonal	TN	Polyclonal	TN	Polyclonal	TN	NEC	TN
00338	FFPE	JMKGY	ID21	Polyclonal	Polyclonal	TN	Polyclonal	TN	Polyclonal	TN	NEC	TN
02189	BM	JMKGY	ID18	Polyclonal	Polyclonal	TN	Polyclonal	TN	Polyclonal	TN	NEC	TN
02961	FFPE	JML7N	ID07	Polyclonal	Polyclonal	TN	Polyclonal	TN	Polyclonal	TN	Clonal	FP
00720	BM	JMKL4	ID14	Polyclonal	Polyclonal	TN	Polyclonal	TN	Clonal	FP	NEC	TN
03101	BLD	JP9L4	ID11	Polyclonal	Polyclonal	TN	Polyclonal	TN	Polyclonal	TN	NEC	TN
01002	FFPE	JMKGY	ID02	Polyclonal	Polyclonal	TN	Polyclonal	TN	Polyclonal	TN	NEC	TN
01859	BLD	JMK3H	ID08	Clonal	Clonal	TP	Clonal	TP	Clonal	TP	Clonal	TP
02942	FFPE	JML7N	ID08	Clonal	Clonal	TP	Clonal	TP	Clonal	TP	Clonal	TP
03040	FFPE	JML7N	ID09	Clonal	Clonal	TP	Clonal	TP	Clonal	TP	Clonal	TP
02817	BM	JML7N	ID19	Clonal	Clonal	TP	Clonal	TP	Clonal	TP	Clonal	TP
02834	FFPE	JMJY7	ID04	Clonal	Clonal	TP	Clonal	TP	Clonal	TP	Clonal	TP
00467	FFPE	JMJY7	ID07	Clonal	Clonal	TP	Clonal	TP	Clonal	TP	Clonal	TP
01539	FFPE	JMKL4	ID16	Clonal	Clonal	TP	Clonal	TP	Clonal	TP	Clonal	TP
02614	BM	JMKL4	ID18	Clonal	Clonal	TP	Clonal	TP	Clonal	TP	Clonal	TP
03060	FFPE	JP9L4	ID10	Clonal	Clonal	TP	Clonal	TP	Clonal	TP	Clonal	TP
02810	FFPE	JML7N	ID18	Clonal	Clonal	TP	Clonal	TP	Clonal	TP	Clonal	TP

*Excluded from analysis

Summary	Invivoscribe Result	MSK Result	NS Result	RP Result
Sum FN	8	8	4	1
Sum TN	10	10	8	8
Sum TP	10	10	14	17
Sum FP	0	0	2	2
Sensitivity	55.6%	55.6%	77.8%	94.4%
Specificity	100.0%	100.0%	80.0%	80.0%
PPV	100.0%	100.0%	87.5%	89.5%
NPV	55.6%	55.6%	66.7%	88.9%

Supplementary Table 3: Results of T cell accuracy for 4 models of clonality results for TRB

Case	Flow Cell	Barcode	T_Invivoscribe	T_Arcila	T_NS	T_RP
02942	JML7N	ID08	Clonal	Clonal	Clonal	Clonal
03040	JML7N	ID09	Clonal	Clonal	Polyclonal	Clonal
00935	JMKL4	ID15	Clonal	Clonal	Polyclonal	Clonal
01542	JML7N	ID11	Clonal	Clonal	Clonal	Clonal
01737	JML7N	ID12	Clonal	Clonal	Clonal	Clonal
01859	JMK3H	ID08	Clonal	Clonal	Clonal	Clonal
03060	JP9L4	ID10	Clonal	Clonal	Clonal	Clonal
00467	JMJY7	ID07	Clonal	Clonal	Polyclonal	Clonal
01228	JMK3H	ID02	Failed	Failed	Clonal	Failed
02319	JMK3H	ID11	Failed	Failed	Polyclonal	Failed
02810	JML7N	ID18	Failed	Failed	Polyclonal	Failed
02189	JMKGY	ID18	Polyclonal	Polyclonal	Polyclonal	NEC
00338	JMKGY	ID21	Polyclonal	Polyclonal	Polyclonal	NEC
01002	JMKGY	ID02	Polyclonal	Polyclonal	Polyclonal	NEC
01464	JP9L4	ID04	Polyclonal	Polyclonal	Polyclonal	NEC
01520	JMK3H	ID05	Polyclonal	Polyclonal	Polyclonal	NEC
02090	JMKGY	ID25	Polyclonal	Polyclonal	Polyclonal	NEC
02961	JML7N	ID07	Polyclonal	Polyclonal	Clonal	Clonal
00720	JMKL4	ID14	Polyclonal	Polyclonal	Polyclonal	NEC
01539	JMKL4	ID16	Polyclonal	Polyclonal	Polyclonal	NEC
02614	JMKL4	ID18	Polyclonal	Polyclonal	Polyclonal	NEC
03599	JMKL4	ID19	Polyclonal	Polyclonal	Polyclonal	NEC
02884	JML7N	ID13	Polyclonal	Polyclonal	Polyclonal	NEC
03101	JP9L4	ID11	Polyclonal	Polyclonal	Polyclonal	NEC
01493	JML7N	ID14	Polyclonal	Polyclonal	Polyclonal	Clonal
02834	JMJY7	ID04	Polyclonal	Polyclonal	Clonal	Clonal
01531	JMKL4	ID22	Polyclonal	Polyclonal	Clonal	Clonal
01659	JMKL4	ID23	Polyclonal	Failed	Clonal	NEC
02029	JP9L4	ID14	Polyclonal	Polyclonal	Clonal	Clonal
02817	JML7N	ID19	Polyclonal	Polyclonal	Polyclonal	NEC
03014	JMKL4	ID27	Polyclonal	Polyclonal	Polyclonal	Clonal
